# Relationship Between Mental Health and Death Anxiety During COVID-19 Pandemic in Dental Staff and Students: A Cross-Sectional Study

**DOI:** 10.3389/fpsyt.2022.849868

**Published:** 2022-05-19

**Authors:** Mahta Jazaiery, Kosar Rezaeifar, Mehdi Sayyah, Maria Cheraghi

**Affiliations:** ^1^Department of Community Oral Health, School of Dentistry, Ahvaz Jundishapur University of Medical Sciences, Ahvaz, Iran; ^2^Department of Oral and Maxillofacial Medicine, School of Dentistry, Ahvaz Jundishapur University of Medical Sciences, Ahvaz, Iran; ^3^Faculty Member of Education Development Center, Psychiatrist, Ahvaz Jundishapur University of Medical Sciences, Ahvaz, Iran; ^4^Department of Community Oral Health, Social Determinant of Health Research Center, School of Dentistry, Ahvaz Jundishapur University of Medical Sciences, Ahvaz, Iran

**Keywords:** COVID-19, mental health, death anxiety, dental staff and students, Iran

## Abstract

**Objective:**

We aimed to determine the mental health and death anxiety among dental staff and students in school of dentistry during COVID-19 pandemic.

**Methods:**

It was a cross-sectional study among students (*n* = 300) and staff (*n* = 60) in School of dentistry in Ahvaz University of Medical Sciences during 2020. The instruments were a demographic questionnaire, Death Anxiety Scale, and Kessler Questionnaire. Data was analyze by using SPSS version 22, in all tests, the significance level was set at <0.05.

**Results:**

The mean age of dental students and personnel was 23.96 and 40.08 years, respectively. The mean scores of death anxiety were higher in dental staff (8.53) than students (6.02) and the mean scores of mental health status were higher in students (14.78) than personnel (9.18). This indicates that death anxiety was higher in Dental staff, while students were in better mental health status. The correlation coefficient between death anxiety and mental health status was 0.366 among students (*p* < 0.001), while it was 0.429 among dental staff (*p* < 0.001), showing a medium relationship between death anxiety and mental health in both groups.

**Conclusion:**

The overall findings represent a significant but contradictory relationship between mental health status and death anxiety among dental staff and students during the prevalence of COVID-19 pandemic. This suggests the impact of confounding factors in this area, which can be studied by future researchers and policy makers to design health promotion interventions.

## Introduction

From the beginning of COVID-19, its rapid outbreak and unknown nature have turned it into a new and critical research topic worldwide (1). This pandemic, as a global health crisis of our time, was considered as the greatest challenge humanity has encountered since World War II (2).

Research findings show that emergence of stressful stimuli at home, such as long-term fear of developing COVID-19 can cause permanent effects on individuals' mental health during the current pandemic (3–5). Due to the contagious nature of this disease, it turned into a pandemic throughout the world causing a high mortality rate as well as great concern and anxiety (6). In health crises and disease outbreaks, Dental staffs are at great risk since they are at forefront of the rapid response group. In early stages of the current pandemic, Dental staff accounted for 29% of all patients admitted to hospitals due to COVID-19 (7). Mental health problems in the Dental staff can cause attention loss, impede cognitive function, and disturb clinical decision-making (8, 9), leading to increased medical errors and accidents that can endanger patients' life (10–12). Dental staff, including the faculty members and students, are easily exposed to viral diseases, such as COVID due to face-to-face contact with patients and exposure to their saliva, blood, and body fluids, and use of sharp tools. Despite the exponential incidence and mortality rates of COVID-19, enough scientific evidences are not available on the psychological effects of this disease on dental staff. Therefore, the present study aimed to investigate the mental health status and death anxiety among the Dental staff and students with regard to COVID-19 outbreak. Our findings can be used by authorities to take appropriate educational, counseling, and treatment measures in order to reduce psychological complications caused by the disease, for this reason we to determine the mental health and death anxiety among dental staff and students in school of dentistry during COVID-19 pandemic.

## Materials and Methods

### Study Location

The study was carried out in Ahvaz Jundishapur University of Medical Sciences which has located in Ahvaz metropolis city in Khuzestan province; southwestern of Iran, with a citizen count of ~1.3 million (3).

### Participants and Implementation Method

It was a descriptive-analytical cross-sectional study was carried out on a total of 360 participants, consisting of dental students (*n* = 300) and Dental staff (*n* = 60) was applied by Census sampling method in 2020–2021. The study participants were selected from all Dental staff and students of the School of Dentistry in Ahvaz Jundishapur University of Medical Sciences. Followed by obtaining the Ethics Code and making the necessary coordination with the faculty dean, the participants were provided with the questionnaire's online link *via* virtual channels and groups attributed to the faculty of dentistry. Inclusion criteria were being employed or studying at the School of Dentistry during the study period, and responding to the questionnaire completely. Furthermore, individuals' unwillingness to continue cooperation at any stage of the research was considered as the exclusion criteria.

### Research Tools

To collect the study data, the following instruments were administered: demographic information questionnaire, Death Anxiety Scale (DAS), and Kessler questionnaire.

The demographic questionnaire included 6 items about the participants' age, gender, marital status, educational level, job position, and working ward.

The Templer DAS was used to assess the participants' death anxiety. This questionnaire consists of 15 correct (0 score)/incorrect (1 score) items and the total attainable scores can range from 0 (absence of death anxiety) to 15 (very high levels of death anxiety). The validity and reliability of DAS was corroborated in the literature with a Cronbach's alpha = 0.73 (13, 14).

The Kessler-K6 Questionnaire contains six items measuring the signs and symptoms of depression during the past month with regard to the respondents' feelings of sadness, nervousness, restlessness, frustration, effort, and worthlessness. The respondents are required to answer based on a 5-point Lickert scale including “always,” “most of the time,” “sometimes,” “rarely,” and “not at all” options. The attainable scores from this questionnaire can range from 6 to 30 showing severe (scores 6–14), moderate (scores: 15–22), and mild (scores: 23–30) levels of anxiety and depression. The reliability and validity of this scale was confirmed in previous studies by a Cronbach's alpha = 0.86 (15, 16).

### Ethical Considerations

In order to observe ethical considerations, all research participants were ensured about confidentiality of their information. To this end, all questionnaires were coded anonymously. The Ethics Code was also achieved from Ahvaz Jundishapur University of Medical Sciences (IR.AJUMS.REC.1399.177).

### Data Analysis

Data analysis was performed using SPSS software version 22 at two descriptive and inferential levels. Descriptive statistics included mean, standard deviation, and frequency distribution. Independent *t*-test, correlation coefficient and variation analysis were also run at the inferential level. The significance level of all tests was considered as <0.05.

## Results

The mean age of students and Dental staff was 23.96 ± 3.04 and 40.08 ± 5.82 years, respectively. The highest frequency of students was related to the third-year students (27%), while their lowest frequency was attributed to the first- and second-year students (4.3%). Furthermore, we found that variables of death anxiety and mental health status had no significant relationship with demographic variables (including age, gender, marital status, academic year, age groups, and province of residence) (Table 1).

**Table 1:** Mean scores of death anxiety and mental health status in dental students and dental staff according to their demographic characteristics.

	**Demographic**				**Death anxiety**	***p*-value**	**Mental health**	***p*-value**
**Participants**	**characteristics**		**Frequency**	**Percent**	**mean score**	**death anxiety**	**mean score**	**mental health**
Dental staff	Age groups	20–30	7	11.7	10.2	0.79	7.91	0.56
		31–40	23	38.3	8.42		8.6	
		41–50	22	36.7	7.89		8.2	
		Over 50	8	13.3	7.74		10.37	
	Gender	Men	23	38.3	7.78	0.691	7.3	0.087
		Women	37	61.7	8.38		10.35	
	Marital status	Single	13	21.7	7.75	0.465	10.15	0.56
		Married	47	78.3	8.72		8.91	
	Occupation groups	Personnel of the dentistry school	30	50	7.17	0.168	9.75	0.997
		Faculty member of periodontics	4	6.7	8.75		6	
		Faculty member of prosthodontics	3	5	11.67		5	
		Faculty member of endodontics	2	3.3	12		9	
		Faculty member of orthodontics	4	6.7	11		9.25	
		Faculty of restorative dentistry	4	6.7	10.75		10	
		Faculty member of oral health	3	5	9.33		10	
		Faculty of oral and maxillofacial surgery	1	1.7	1		6	
		Faculty member of oral pathology	1	1.7	12		7	
		Faculty member of oral and maxillofacial radiology	1	1.7	8		9.67	
		Faculty member of oral and maxillofacial diseases	3	5	8.67		9	
		Faculty member of pediatric dentistry	4	6.7	10.25		9.75	
Students	Age groups	18–20	19	6.3	5.63	0.222	16.11	0.124
		21–30	267	89	5.93		14.47	
		31–40	13	4.4	8.31		19.85	
		Over 40	1	0.3	7		8	
	Gender	Men	122	40.7	5.92	0.731	13.64	0.6
		Women	178	59.3	6.08		15.57	
	Marital status	Single	261	87	5.81	0.023	14.72	0.732
		Married	39	13	7.41		15.23	
	Academic year	1 year	13	4.33	5.38	0.741	14.23	0.538
		2 year	13	4.33	6.15		14.23	
		3 year	85	27	6.6		16.04	
		4 year	67	22.34	5.96		14.6	
		5 year	53	17.67	5.58		11.81	
		6 year	73	24.33	5.82		15.92	

Findings from comparisons between the study groups showed that death anxiety was higher in dental staff, while students were in better mental health status. The correlation coefficient between death anxiety and mental health status was 0.366 among students (*p* < 0.001), while it was 0.429 among dental staff (*p* < 0.001), showing a medium relationship between death anxiety and mental health in both groups (Table 2).

**Table 2:** Mean scores and correlation coefficient between death anxiety and mental health status in students and dental staff.

**Variables**		**Mean**	**Standard deviation**	**Correlation coefficient**	***p*-value**
Death anxiety	Dental staff	8.53	3.70	0.429	*p* < 0.001
Mental health status	Dental staff	9.18	6.70		
Mental health status	Students	6.02	4.10	0.366	*p* < 0.001
	Students	14.78	7.73		

## Discussion

The present study aimed at determining the mental health status and death anxiety among the Dental staff and students during COVID-19 pandemic. In students, the mean score of death anxiety and mental health status was 6.02 and 14.78, respectively. In the Dental staff, the mean score of death anxiety and mental health status was 8.53 and 9.18, respectively. Higher scores of mental health status in students indicate their critical and inappropriate psychological condition.

The correlation coefficient between the two variables of death anxiety and mental health status was 0.366 among dental students (*p* < 0.001). Given the value of Spearman correlation coefficient (0.366), a strong significant correlation was found between death anxiety and mental health status among students. In line with these results, White et al. reported that people with high death anxiety had significantly higher levels of mental health status compared to people with low death anxiety (17). The correlation coefficient between death anxiety and mental health status was 0.429 among Dental staff (*p* < 0.001), showing a significant relationship between these two variables. The Spearman correlation coefficient value of −0.429 indicates a medium correlation between death anxiety and mental health status, but the negative sign indicates the inverse relationship of death anxiety score with mental health status in Dental staff. In other words, Dental staff with higher death anxiety scores had lower mental health status scores. The fact that death anxiety showed a significant negative relationship with mental health status implies that people who reported less psychological symptoms (such as stress) had higher death anxiety. This discrepancy may indicate that the etiology of death anxiety may have a variety of environmental, familial, biological, and social causative factors. In other words, some confounding variables may have played role in this correlation. These findings are consistent with other studies (18), but are different from those reported by Belsky et al., Stevens et al., and Masoudzadeh et al. (19–21). Among the most important reasons for students' anxiety, worrying about the effect of COVID-19 on their future education, future job status, and reduced social relationships can be mentioned (22). Anxiety in some students may be due to problems in providing university tuition caused by loss of financial resources followed by unemployment and job loss (23). Acute conditions of COVID-19 have also caused important psychological disorders such as mental health status, anxiety, and symptoms of mental disorders among Dental staff (24). The fact that Dental staff should stay away from their family members in the pandemic conditions may affect their mental health. Even in the case that Dental staff visit their family, they may experience extreme anxiety and fear about transmitting the disease to their family members (22). They can also develop similar psychological disorders caused by the fear of being infected by COVID-19 *via* patients and clients who refer to the health care centers (22, 24, 25).

Continuation of COVID-19 pandemic has exposed many people to death anxiety and low mental health status causing great concerns in the health care community. Consequently, scientific interventional programs are required to reduce psychological complications of COVID-19 among the Dental staff and students.

## Conclusion

According to the findings, a significant but contradictory relationship was observed between mental health status and death anxiety among the Dental staff and students in COVID-19 pandemic. This indicates the impact of confounding factors in this area, which can be investigated by future researchers and planners to design health promotion interventions. Also, dental staff and students may receive less attention in the current high-risk situation under influence of the highly contagious COVID-19. Therefore, appropriate remote methods of psychotherapy should be applied *via* video conferencing and online programs using appropriate applications or even phone calls to treat anxiety, depression, and post-traumatic stress disorder among the Dental staff.

### Study Limitations

Given that the study questionnaires were completed based on the respondents' self-report information, their responses may have been affected by their psychological status that could have affected the study results. Moreover, the sample size and research population of the present study may have limited generalizability of the findings.

## Data Availability Statement

The raw data supporting the conclusions of this article will be made available by the authors, without undue reservation.

## Ethics Statement

The studies involving human participants were reviewed and approved by Ahvaz Jundishapur University of Medical Sciences Ethics Committee. Written informed consent for participation was not required for this study in accordance with the national legislation and the institutional requirements.

## Author Contributions

MC and KR were principal investigators of the study. MC and MS were advisors of the study. MJ was collected the data and drafted the manuscript. MC performed the statistical analysis. All authors contributed to the design, data analysis and assisted in the preparation of the final version of the manuscript, and read and approved the final version of the manuscript.

## Funding

This study was as part of dissertation by Ms. Mahtab Jazaieri in School of Dentistry. This study was supported by Social Determinants of Health Research Center in Ahvaz Jundishapur University of Medical Sciences, Ahvaz, Iran (SDH-9906).

## Conflict of Interest

The authors declare that the research was conducted in the absence of any commercial or financial relationships that could be construed as a potential conflict of interest.

## Publisher's Note

All claims expressed in this article are solely those of the authors and do not necessarily represent those of their affiliated organizations, or those of the publisher, the editors and the reviewers. Any product that may be evaluated in this article, or claim that may be made by its manufacturer, is not guaranteed or endorsed by the publisher.
